# Associations Between Endogenous Estrogen, Postmenopausal Hormone Therapy, and Cognitive Changes in Older Women

**DOI:** 10.1093/geroni/igab046.1816

**Published:** 2021-12-17

**Authors:** Mark Espeland, Christina Hugenschmidt

**Affiliations:** Wake Forest School of Medicine, Winston-Salem, North Carolina, United States

## Abstract

How markers of brain health are associated with endogenous estrogen and use of postmenopausal hormone therapy (HT) varies depending on women’s years from menopause and metabolic health status, ranging from potential benefit to harm. The Women’s Health Initiative (WHI) included 7,233 women age 65-80 who underwent a randomized clinical trial of various HT preparations for an average of 5.9 years. Over up to 18 years of post-trial follow-up, diabetes (DM2) increased the risk of dementia (hazard ratio [HR] 1.54 [95% CI 1.16–2.06]). Having DM2 and also treatment with unopposed conjugated equine estrogens increased the risk to HR=2.12 [1.47-3.06]. We hypothesize that the metabolic effects of estrogen in the brain drives this interaction. In support of this, the metabolic transition following menopause may alter the impact of other treatments on cognition, for example behavioral weight loss therapy to treat obesity in women with type 2 diabetes (interaction p=0.02 for executive function).

